# Computer-Aided Detection (CADe) System with Optical Coherent Tomography for Melanin Morphology Quantification in Melasma Patients

**DOI:** 10.3390/diagnostics11081498

**Published:** 2021-08-19

**Authors:** I-Ling Chen, Yen-Jen Wang, Chang-Cheng Chang, Yu-Hung Wu, Chih-Wei Lu, Jia-Wei Shen, Ling Huang, Bor-Shyh Lin, Hsiu-Mei Chiang

**Affiliations:** 1 Apollo Medical Optics, Ltd., Taipei City 11491, Taiwan; esther.x10@gmail.com (I.-L.C.); cwlu@mdamo.com (C.-W.L.); lynn@mdamo.com (L.H.); 2 Department of Dermatology, MacKay Memorial Hospital, Taipei City 10449, Taiwan; yenjen4208@gmail.com (Y.-J.W.); dr.yhwu@gmail.com (Y.-H.W.); 3 Department of Cosmetic Applications and Management, MacKay Junior College of Medicine, Nursing, and Management, New Taipei City 25245, Taiwan; 4 Institute of Imaging and Biomedical Photonics, National Yang Ming Chiao Tung University, Tainan 71150, Taiwan; borshyhlin@nctu.edu.tw; 5 Department of Cosmeceutics, China Medical University, Taichung 40433, Taiwan; winnie953444@yahoo.com.tw (J.-W.S.); hmchiang@mail.cmu.edu.tw (H.-M.C.); 6 School of Medicine, College of Medicine, China Medical University, China Medical University Hospital, Taichung 404332, Taiwan; 7 Aesthetic Medical Center, China Medical University Hospital, Taichung 40402, Taiwan; 8 Department of Medicine, Mackay Medical College, New Taipei City 25245, Taiwan

**Keywords:** melasma, melanin, optical coherence tomography, cellular resolution, full-field OCT, photoaging, deep learning, image denoising, convolutional neural networks, computer-aided detection

## Abstract

Dark skin-type individuals have a greater tendency to have pigmentary disorders, among which melasma is especially refractory to treat and often recurs. Objective measurement of melanin amount helps evaluate the treatment response of pigmentary disorders. However, naked-eye evaluation is subjective to weariness and bias. We used a cellular resolution full-field optical coherence tomography (FF-OCT) to assess melanin features of melasma lesions and perilesional skin on the cheeks of eight Asian patients. A computer-aided detection (CADe) system is proposed to mark and quantify melanin. This system combines spatial compounding-based denoising convolutional neural networks (SC-DnCNN), and through image processing techniques, various types of melanin features, including area, distribution, intensity, and shape, can be extracted. Through evaluations of the image differences between the lesion and perilesional skin, a distribution-based feature of confetti melanin without layering, two distribution-based features of confetti melanin in stratum spinosum, and a distribution-based feature of grain melanin at the dermal–epidermal junction, statistically significant findings were achieved (*p*-values = 0.0402, 0.0032, 0.0312, and 0.0426, respectively). FF-OCT enables the real-time observation of melanin features, and the CADe system with SC-DnCNN was a precise and objective tool with which to interpret the area, distribution, intensity, and shape of melanin on FF-OCT images.

## 1. Introduction

Dark skin-type individuals have more hyperactive melanocytes compared to the fair skin-type individuals and are prone to develop melasma and other pigmentary disorders [1,2,3]. The deposit of melanin in keratinocytes is readily seen on hematoxylin and eosin stain (H&E stain), while melanocytes must be identified by special staining. With the emerging non-invasive techniques, melanin in the epidermis can be easily visualized because of its brightness by dermatofluoroscopy [4], reflectance confocal microscopy (RCM) [3], multiphoton microscopy [5], and optical coherence tomography (OCT) [6]. Studies suggested melanocytes rapidly transfer the produced melanin to keratinocytes rather than accumulating it [7]. This may explain that typical active dendritic melanocytes were hardly observed, while supranuclear melanin caps in the keratinocytes were easily visible [8,9]. Melanin amount and distribution are therefore commonly used to classify the melasma subtypes and to monitor the treatment response [10]. Non-invasive techniques including RCM and multiphoton microscopy already detect pigmentary changes after treatment with the picosecond alexandrite laser and Q-switched Nd: YAG laser [3,5,10,11]. We recently demonstrated the cellular resolution full-field optical coherence tomography (FF-OCT) system allows real-time, non-invasive imaging of superficial skin diseases [6]. Although these imaging techniques allow observation of in vivo melanolysis and qualitative pigmentary changes, studies with quantitative measurements of the amount and intensity of melanin remain scarce [4,12,13]. On the other hand, the images are often taken on multiple different areas on the face and across the timeline of months. This ultimately results in a large number size of images for physicians to interpret. Aside from the labor, human eyes are prone to bias and weariness and may not be able to detect the delicate pigmentary changes. To address the above obstacles encountered, we propose a computer-aided detection (CADe) system with denoising, which can automatically segment and mark melanin on FF-OCT images and provide more objective, reliable melanin descriptions and analysis of its distribution in different skin layers for diagnosis through quantified morphological features.

## 2. Materials and Methods

**Figure 1:**
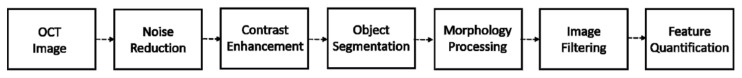
Block diagram of the proposed computer-aided detection (CADe) system.

FF-OCT is an optical interferometric technique for high-resolution 3D imaging. Yet, the OCT images inherently encounter the speckle noise, and hence the image quality is reduced. Spatial compounding (SC) is a commonly used technique to mitigate the speckle and Gaussian noise. The principle of SC is to induce changes in the speckle pattern between repeated measurements through the tiny position change of the subject. Then, these partially de-correlated multiple images measured from the sample are averaged to obtain low-speckle images. Previous studies have confirmed the denoising performance of SC in ophthalmic images [14,15]. In particular, through FF-OCT, multiple adjacent scans can be acquired simultaneously without additional registration effort for SC. However, the tradeoffs are greater time consumed, motion blur, and potential reduction of spatial resolution. To address the difficulties, preliminary research [16] combined SC and deep learning to provide effective noise reduction while maintaining the details of the OCT image. An SC-based denoising convolutional neural network (SC-DnCNN) is used in the presented CADe system to improve image quality. The automatic procedure of the CADe system is shown in Figure 1. First, a denoising convolutional neural network (CNN) model is employed to remove speckle noise from the tissue on images. Then, a series of image processing actions, including contrast enhancement, object segmentation, morphology processing, and image filtering, are used to automatically detect complete melanin-related objects (including melanin, melanosome, melanocyte, and melanophage) in OCT images and even in different skin layers. Finally, various melanin-related characteristics are extracted from detected objects as the quantitative features for diagnosis.

### 2.1. Patients and Data Acquisition

The FF-OCT scanner from ApolloVue^®^ S100 Image System (manufactured by Apollo Medical Optics, Ltd., Taipei City, Taiwan) was used to collect the skin OCT image (897 × 899 pixels, about 0.5 μm/pixel image resolution) and store it with 8-bit pixel depth. All OCT images used in this research were obtained between August 2020 and November 2020 from eight Asian patients with moderate to severe melasma from Taichung, which is located in subtropical area in Taiwan. The MASI score of the 8 patients ranged 11.95–16.70 (mean 14.09 ± 1.73). Half of these patients were Fitzpatrick skin type III, and half were skin type IV, with their ages ranging from 51 to 65 (median: 56).

Two lesion fields of view (FOVs) and one perilesional skin FOV were taken from the left and right cheeks of each patient. There were 96 lesion images and 48 perilesional skin images that contained three layers—the *en face* stratum spinosum, the dermal–epidermal junction (DEJ), and papillary dermis—for the experiment.

### 2.2. SC-DnCNN

**Figure 2:**
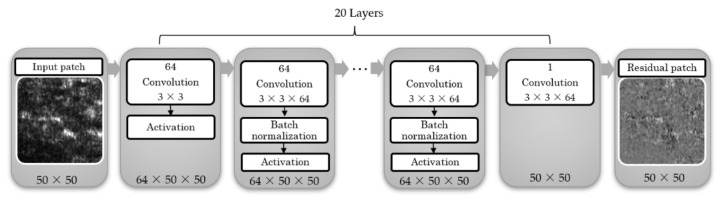
The deep learning architecture of the denoising convolutional neural network (DnCNN).

The SC-DnCNN is a pixel-wise noise prediction method that can be used to distinguish the noise in the signal, thereby improving the image quality. It follows the advantages of a denoising convolutional neural network (DnCNN) [17], taking residual learning [18] and batch normalization (BN) [19] to speed up the training process and improve the denoising performance. As shown in Figure 2, the deep architecture of a DnCNN is based on the concept of the visual geometry group (VGG) network [20] and consists of multiple smaller convolutional layers. The composition of these layers can be divided into three main types. The first type appears in the first layer. It uses 64 filters with a size of 3 × 3 to generate 64 feature maps and then performs nonlinear conversion through rectified linear units (ReLU) [21] on these feature maps as the input to the next layer. From the second layer to the penultimate layer, all these convolutional layers belong to the second type. Similarly, 64 filters with a size of 3 × 3 × 64 are used on the input maps, but unlike the previous layer, BN is added before ReLU. The BN is a normalization method that adjusts the distribution of input values to a normal distribution, which not only avoids the problem of gradient vanishing but also greatly accelerates the training speed. Finally, a filter with a size of 3 × 3 × 64 is used in the last layer as the output reconstruction.

**Figure 3:**
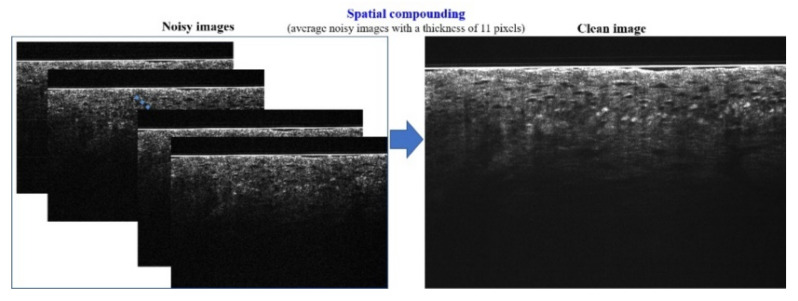
Schematic diagram of how to generate low-speckle ground truth images.

In model training, the residual learning concept of deep residual network (ResNet) is applied to simplify the optimization process. The difference is that DnCNN does not add a shortcut connection between several layers but directly changes the output of the network to a residual image. This means that the optimization goal of DnCNN is not the mean square error (MSE) between the real clean image and the network output but the MSE between the real residual image and the network output. The residual image, the noise map, could be obtained by subtracting the clean image from the noisy image. In the previous study [17], the noise is randomly added to a clear image to simulate a noisy image. Regarding OCT images, the noise is mainly composed of the speckle noise, which multiplies the noise by the structure signal [22]. Therefore, we generate the ground truth by using real OCT images rather than simulated ones. Figure 3 illustrates the method of SC-based ground-truth generation. In our FF-OCT imaging system, 11-pixel lines are activated to acquire cross-sectional view (B-scan) images; accordingly, 11 adjacent virtual slices are generated for SC. The thickness of the compounding image is around 5 μm, which is close to histological slices. As the clean image, the composite image with low speckle is obtained by averaging 11 adjacent B-scans. In contrast, the average image generated by compounding *N* pixel lines outward from the center represents a noisy image (where *N* < 11).

**Figure 4:**
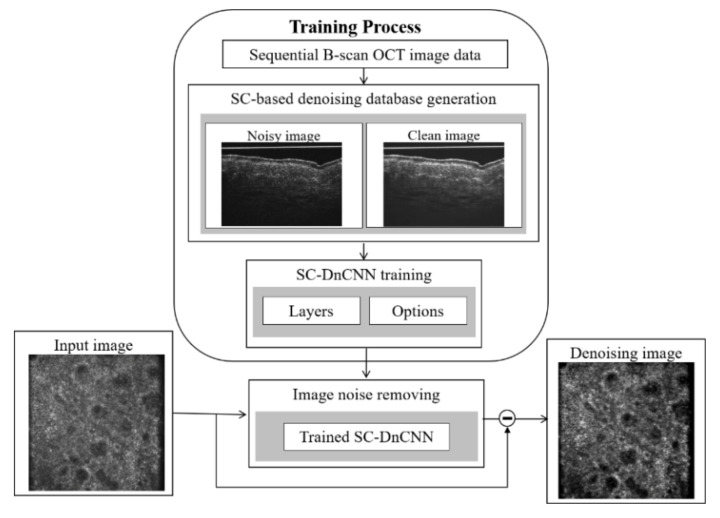
The structure of the spatial compounding-based denoising convolutional neural networks (SC-DnCNN) trained for optical coherence tomography (OCT) denoising.

The training and implementation structure of the SC-DnCNN model are shown in Figure 4. According to the successful demonstration of B-scan image denoising in the previous study [16], we chose a model trained with noisy images compounded by 5-pixel lines to improve the *en face* scan (E-scan) image quality in this work. To train the SC-DnCNN model, 512 paired patches with a size of 50 × 50 were randomly cropped in each pair of images (noisy image and noise map). We set the number of network layers to 20 and used the stochastic gradient descent method to automatically learn the weights of the filter kernels. In this deep learning, the parameter settings for model training, including momentum, learning rate, mini-batch size, and epochs, were 0.9, 0.001, 128, and 50, respectively. With reference to previous related studies [17,23], for the training dataset of the denoising model, only about 300–400 images are needed to achieve the outstanding effect, and increasing the amount of training data has a small improvement in performance [17]. In our research, the model was trained and verified via 335 B-scan OCT images collected from 10 patients [16]. The specifications of all B-scan data captured with the whole FF-OCT scan were 1024 × 715 pixels, about 0.5 μm/pixel image resolution, and storage in 8-bit pixel depth.

### 2.3. Melanin Detection

The compartment of melanin taken up by keratinocytes from melanocytes was recently given the name melanokerasome [7,8,24,25,26,27]. Our previous study comparing hematoxylin and eosin (H&E) stain slice and OCT images revealed melanin appears as hyper-reflective cells with a relatively strong intensity compared with the surrounding tissues [6]. To replace the manual marking of melanin, a series of image processing steps based on signal brightness and shape are proposed as follows.

**Figure 5:**
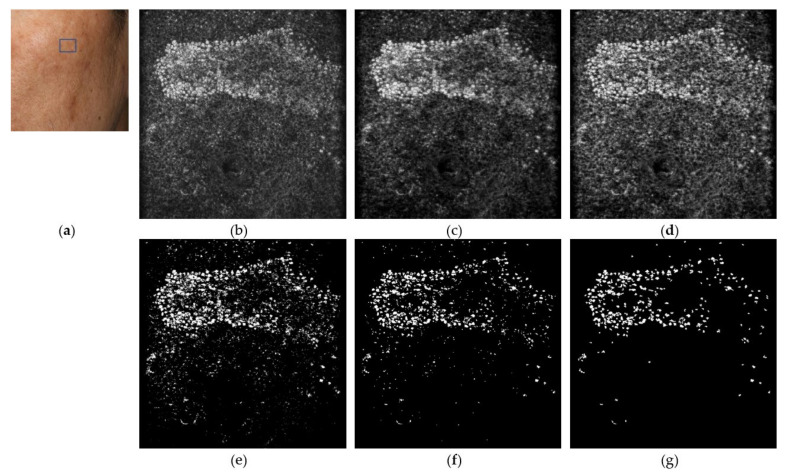
Illustration of automatic melanin segmentation in CADe system. (**a**) Clinical image of the imaged melasma lesions (rectangular) on the cheek. (**b**) The original *en face* scan (E-scan) image. (**c**) The image after SC-DnCNN. (**d**) The image after performing contrast-limited adaptive histogram equalization (CLAHE). (**e**) The candidate targets segmented by thresholding. (**f**) The selected grain melanin after image opening. (**g**) The selected confetti melanin after morphological operations. The field of view is 475 × 476 µm.

Here, we take the E-scan OCT image in Figure 5 as an example to describe the complete melanin segmentation process. First, we generate a clear image in Figure 5b by performing noise reduction through the SC-DnCNN model. Second, contrast-limited adaptive histogram equalization (CLAHE) [28] is applied to stretch the contrast in each local area (approximately 12.5 ×12.5 μm tile) to enhance the feature of melanin whose intensity is stronger than the surrounding signal, as shown in Figure 5c. Several specified parameters in CLAHE, including the number of tiles into which the image is divided, the distribution type for creating the contrast transform function, and the limiting factor that controls the contrast enhancement effect, are determined through experiments to be 40 × 40, exponential (λ = 0.1), and 0.001, respectively. Next, a relatively loose brightness level with a threshold of 0.6 is given to filter out the target whose local signal does not reach a certain intensity, which means that all pixels in the enhanced image that exceed the 153 gray level are regarded as candidates for melanin. The result of binarization segmentation is shown in Figure 5d. Finally, according to the observation results of the melanin size in the electron microscope in the literature [29] and the limitation of OCT on the resolution of melanin imaging, all targets with a diameter greater than 0.5 mm are retained in Figure 5e after the image opening is applied. In addition, considering the differences in the aggregation forms of melanin, we further performed morphological operations on the binarized image to explore targets with a certain degree of aggregation. As shown in Figure 5f, all the objects with an area over 8.42 μm^2^ (about a circle with a diameter of 3.3 μm) are defined as confetti melanin.

### 2.4. Feature Quantification

According to the electron microscopy study of the distribution pattern, morphology, and size of melanosomes in keratinocytes [7,8,24,25,26,27], melanosome size revealed a progressive variation in size with ethnicity. Melanosomes in dark skin were the largest, followed by those in Asian skin and Caucasian skin [5,9,29]. In dark skins, melanosomes have larger pigment cores and are individually distributed throughout the cytoplasm of keratinocytes, whereas in light skins, they have smaller cores and are aggregated in clusters [7,8,25,26]. Following these quantified findings, all quantitative features collected from OCT images were employed in our CADe system to analyze the state of melanin.

Based on the analysis of melanin-related objects with different forms, the quantitative features extracted from the segmented targets can be classified into two groups: grain and confetti melanin features. The grain melanin features are used to describe all melanin-related objects with a diameter greater than 0.5 μm, while the confetti melanin features symbolize the appearance of all melanin-related objects with a certain degree of connection and an area greater than 8.42 μm^2^. Among them, all confetti melanin belong to grain melanin. A total of 18 quantitative features, including area, distribution, brightness, and shape, are listed in Table 1. The area-based features separately count the total area of all grain melanin and confetti melanin segmented from an image. The distribution-based feature of all grain melanin, *G_density*, is based on the total area of the tissue in the image to calculate the proportion of its area, where the tissue is defined as the signal whose grayscale value is greater than 38 in the enhanced image. The distribution-based features of all confetti melanin are related to their distance in two-dimensional space. *C_distance_mean* and *C_distance_SD*, respectively, use the centroid of each confetti melanin to compute the average and standard deviation of the distance between each other. In addition, the features based on shape and brightness, respectively, provide statistical information to determine the size and intensity of all melanin in the image. To extract the *C_roundness* feature, a simple metric indicating the roundness of confetti melanin is defined as

(1) $$\mathrm{roundness}\;\;\frac{4\pi *area}{perimeter^{2}}$$

where *perimeter* and *area* are the total number of confetti melanin contour pixels and area pixels, respectively.

### 2.5. Statistical Analysis

To explore the correlation between melasma and melanin, the potential of quantitative features in distinguishing lesion images from perilesional skin images was evaluated by several statistical hypothesis tests. For comparison, all data before and after the image denoising were also tested to observe the effect of the SC-DnCNN model on the CADe system. Whether there was a normal distribution of the feature was determined by the Kolmogorov–Smirnov test [30]. Subsequently, the difference of each feature between the lesion and perilesional skin cases was evaluated with the mean ± SD in a normal distribution and the median in a non-normal distribution by using Student’s *t*-test [30] and the Mann–Whitney *U* test [30], respectively. With the significance analysis, *p*-value of less than 0.05 indicated the difference was significant.

## 3. Results

**Figure 6:**
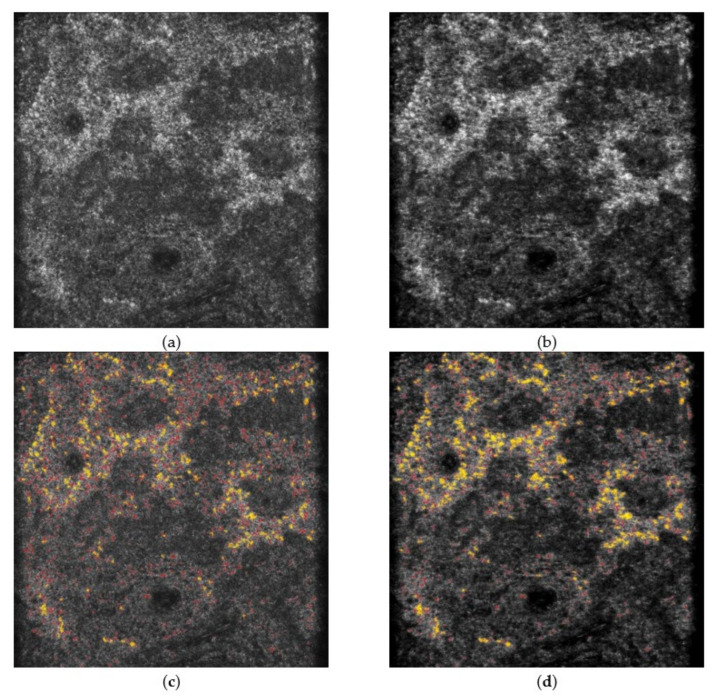
The performance comparison of CADe with and without SC-DnCNN on the representative lesion images. (**a**,**b**) are the input image and the denoised image, respectively, while (**c**,**d**) are the output results when superimposing the detected melanin on those images (grain melanin is represented in red, and confetti melanin is represented in yellow), respectively. The field of view is 475 × 476 µm.

**Figure 7:**
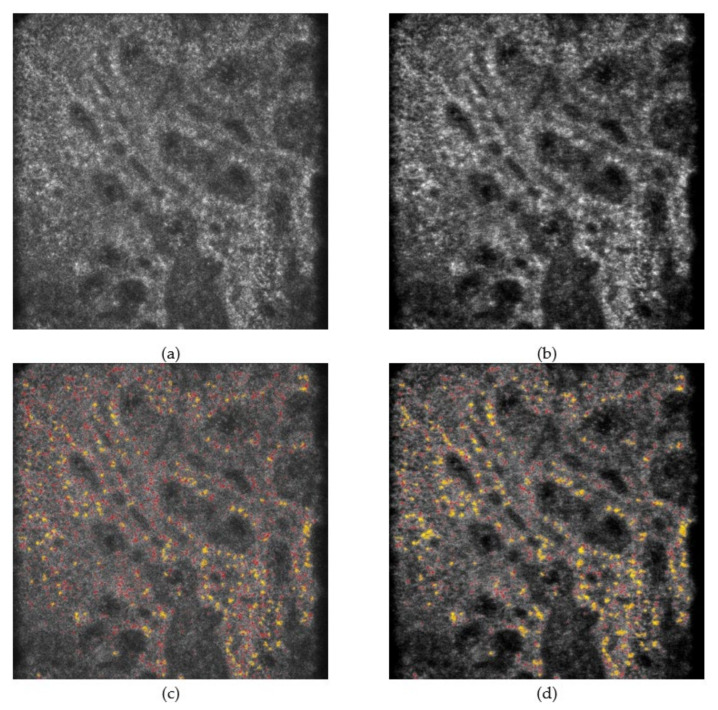
The performance comparison of CADe with and without SC-DnCNN on the perilesional skin images. (**a**,**b**) are the input image and the denoised image, respectively, while (**c**,**d**) are the output results when superimposing the detected melanin on those images (grain melanin is represented in red, and confetti melanin is represented in yellow), respectively. The field of view is 475 × 476 µm.

These experiments verified the performance of the CADe system and SC-DnCNN with a total of 144 images. For all images processed by SC-DnCNN, the noticeable speckle noise was reduced, and the sharpness of the skin tissue structure was also improved. These effects not only help the observation of image details but also show obvious advantages for CADe’s melanin recognition ability. Comparing the results of the CADe system with and without SC-DnCNN, the former showed more precise detection effects, including subtle local contrast changes in the presence of grain melanin and obvious aggregation of confetti melanin. Figure 6 and Figure 7 show the test results for representative lesion and perilesional skin images, respectively. When the influence of noise in the background was reduced, the observation and detection of melanin became clearer and easier.

Table 2 and Table 3 list the performance difference of the proposed CADe system when performed with and without SC-DnCNN. The *p*-values and mean ± SD of all distinct features generated before and after image denoising were extracted and analyzed. Table 2 shows that the *C_distance_mean*, a feature representing the average distance of each centroid of all confetti melanin, differs markedly between lesions and perilesional skin (*p* = 0.0402) with denoising. The average distances of confetti melanin in perilesional skin and lesion images computed by the CADe were 200 μm and 193.5 μm, respectively, while they were 206.1 μm and 200.3 μm, respectively, for CADe without SC-DnCNN. The value of the *C_distance_mean* in the lesion image tended to be smaller than that of the perilesional skin image. However, the difference was not statistically significant (*p* = 0.0502) when image denoising was not performed.

**Figure 8:**
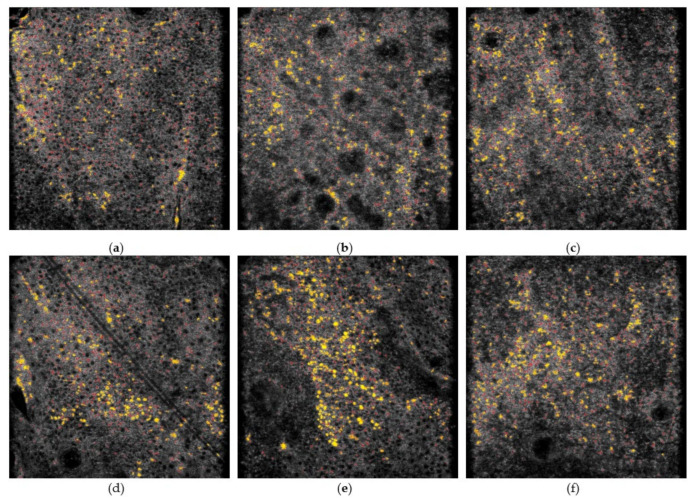
Comparison of melanin appearance between the representative lesion and perilesional skin images in different skin layers displayed by the CADe system. (**a**–**f**) are the perilesional skin images and the lesion images in the stratum spinosum, dermal–epidermal junction (DEJ), and papillary dermis, respectively. The field of view is 475 × 476 µm.

Additionally, we divided the dataset into three subsets according to the skin layer (stratum spinosum, DEJ, and papillary dermis) and evaluated the difference between the melanin features that could distinguish lesions in each subset. Figure 8 shows the appearance of melanin observed in the representative lesion and perilesional skin images of different skin layers using CADe. The *p*-values and mean ± SD of different features generated before and after image denoising for each subset are also summarized in Table 3. In the stratum spinosum, both significant features symbolize the distribution of the confetti melanin, where the larger the *C_distance_mean* is, the more dispersed the melanin will be. In addition, the smaller the *C_distance_SD* is, the more evenly distributed the melanin in the entire image will be. This means that compared with the perilesional skin, the distribution of confetti melanin in the lesion is more clustered in the local area of the image. The *p*-values of *C_distance_mean* and *C_distance_SD* were 0.0036 and 0.0202, respectively, before image denoising, while they were 0.0032 and 0.0312, respectively, after image denoising. Without executing image denoising, all the quantitative features of the DEJ and papillary dermis were not significantly different between the lesion and the perilesional skin. Through SC-DnCNN, the *p*-value of *G_density* in the DEJ was reduced from 0.1393 to 0.0426. For the lesion images, it indicates that the feature of the grain melanin density tends to be higher than that in the perilesional skin image.

## 4. Discussion

Imaging modalities aid the diagnosis, monitoring, and treatment response for many conditions, especially precancer lesions and skin cancers [6,31,32]. These techniques have further expanded the field of cosmetic dermatology in detecting clinically undetectable cell changes of the skin, affording new insights into the mechanisms and kinetics of pigmentary disorders [3,10,33,34]. Although FF-OCT provides dermatologists real-time cellular images, naked-eye evaluation is subject to weariness and inter- and intra-observer variability. It is also time-consuming for dermatologists to perform a complete skin screening. Therefore, many new technologies have this technology integrated into the device, or it is upcoming as a potential future direction to aid in diagnostic accuracy.

In this research, a CADe system has been proposed to automatically label and quantify melanin in FF-OCT images. Combined with the SC-DnCNN, the differences in melanin features between the lesion and the perilesional skin are more distinct. The experiments with 96 lesion and 48 perilesional cases showed that the proposed CADe system helps to distinguish lesions using melanin features. For all images, the denoising performance achieved similar performance so that the overall skin tissue structure was clearer and the melanin pattern to be observed was more complete. Since FF-OCT has a nearly isotropic resolution in all three dimensions, even though the denoising model was trained using 235 B-scan images, it works well on E-scan images. Moreover, because the SC-DnCNN removes the speckle noise correlated with the microstructure of the tissue in the image while performing the automatic detection of melanin, the subsequent series of signal intensity processing actions can be more effective. Through our CADe system, it is possible to quantitatively evaluate and compare some melanin characteristics belonging to the lesion, including its appearance in different skin layers. Moreover, the experimental results show that when diagnosing lesions, different skin layers should emphasize different types of melanin features. When observing the OCT images within a lesion, the confetti melanin appears dense and concentrated in the stratum spinosum, while the grain melanin has a higher density in the DEJ. This is consistent with the findings of previous research [9]. Different skin layers produce different forms of melanin, and their appearance on OCT images is also different. Within keratinocytes, melanokerasomes form a supra-nuclear cap, shielding the nuclear genetic material from ultraviolet radiation-induced damage. Even in our CADe system, a set of universal parameters was successfully applied to the detection algorithm of generalized pigments. Once skin layer information is added, the precision of melanin interpretation can be improved by updating the parameters or conditions of the detection algorithm. Furthermore, useful features in the papillary dermis will have the opportunity to be discovered by excluding other highly reflective skin components such as collagen and keratin.

Measuring and quantifying melanin non-invasively is beneficial for lesion diagnosis and follow-up. The stratum corneum of skin could be harvested by tape-stripping, followed by analysis of melanin content with high-performance liquid chromatography (HPLC) [35]. The accuracy of HPLC is widely accepted; however, sampling only on the stratum corneum layer may not have sufficient information or be a late indicator for diagnosis. Using NIR fluorescence spectroscopy could specify melanin by analyzing the corresponding spectrum and relate the optical signal intensity to melanin concentrations [36]. Lack of structure information of skin, including the distribution of melanin, the size of melanin, and thickness of skin layers, could limit the efficacy of diagnosis. The multispectral imaging device combines the auto-fluorescence signal and narrow-band imaging to extract the spectral information in the images [37]. However, it still lacks the melanin distribution in the skin and the size of melanin. In this study, FF-OCT provides high-resolution three-dimensional images, which could allow us to classify melanin into different sizes and analyze them in different depths of the skin. The results show it does contribute to distinguishing melasma and perilesional skin.

There were some limitations of this study. In our experiments, not all melanin displayed in OCT images had a clear outline. There were individual cases where it overlapped or could not be segmented exactly because aggregation affected the calculation of morphology. For this reason, the shape-based features for confetti melanin, including roundness and size-related symbols, did not play a role in distinguishing the lesion from the perilesional skin. Clinically, the characteristics of melanin, including area, distribution, intensity, and shape, are often used to interpret the lesion on FF-OCT images. However, it is a challenge for CADe to be used on a 2D OCT image. According to the experimental results, the depth information of the skin can support the CADe system’s more effective processing of highly heterogeneous images. This means that the development of melanin detection on three-dimensional images with stereo information will be expected to improve the performance of CADe. By then, the existing ground truth uncertainty will also be better resolved through the continuous information from three-dimensional images. Once the 3D reconstruction of these feature structures existing in the two-dimensional images is completed, the observable melanin three-dimensional morphological features, including shape and distribution, will be more effectively confirmed. Furthermore, the use of more complete feature descriptions will also help to deal with more challenging problems such as lesion classification. Another limitation of this study is that the current research was conducted only for Asians. Yet, although there are differences in the size and concentration of melanin between races, the changes in its optical properties are not easily reflected in the appearance of confetti melanin under limited OCT resolution. Therefore, the applicability of the automatic melanin detection performance to different races, such as Hispanics and Caucasians, can be expected. This is also an important research direction to verify the reliability of CADe in the future.

## 5. Conclusions

FF-OCT has verified the corresponding characteristics of stained sections and has even attracted attention for its ability to diagnose lesions in vivo. Through SC-DnCNN, we can obtain high-quality OCT images and combine them with the proposed CADe system to improve the performance of melanin detection. The CADe system is used to automatically mark melanin-related objects, including grain and confetti form. Various quantitative features can be computed according to the morphology of melanin and used to describe the appearance of the lesions.

In this study, some subtle differences between the lesions and the perilesional skin were revealed with the melanin-related features. These include the distance between confetti melanin regardless of the skin layer, the distribution-based features of confetti melanin in stratum spinosum, and the distribution-based features of grain melanin at the DEJ. Assuming that all pigment diseases have abnormal melanin manifestations, our OCT and algorithm will help capture these differences in patterns and distributions. The proposed CADe system can assist image interpretation and provide effective information about the characteristics of melanin by quickly scanning and marking reminders.

Owing to its capability of reaching real-time and stable detection results, together with its objectivity and precision when describing melanin features, this method could surely represent an attractive tool to address pigment classification problems with such requirements.

## Figures and Tables

**Table 1:** Quantitative features of melanin-related objects on E-scan OCT images.

Form	Category	Feature	Definition
grain	Area	*G_area*	The area of all grain melanin
	Distribution	*G_density*	The density of the grain melanin in the tissue
	Brightness	*G_intensity_min*	The minimum brightness of the grain melanin
		*G_intensity_max*	The maximum brightness of the grain melanin
		*G_intensity_mean*	The average brightness of the grain melanin
		*G_intensity_SD*	The standard deviation of the grain melanin brightness
confetti	Area	*C_area*	The area of all confetti melanin
	Distribution	*C_distance_mean*	The average distance between the centroid of confetti melanin
		*C_distance_SD*	The standard deviation of the distance of confetti melanin centroid
	Shape	*C_roundness*	The average roundness of all confetti melanin
		*C_size_min*	The minimum size of all confetti melanin
		*C_size_max*	The maximum size of all confetti melanin
		*C_size_mean*	The average size of all confetti melanin
		*C_size_SD*	The standard deviation of the confetti melanin size
	Brightness	*C_intensity_min*	The minimum brightness of the confetti melanin
		*C_intensity_max*	The maximum brightness of the confetti melanin
		*C_intensity_mean*	The average brightness of the confetti melanin
		*C_intensity_SD*	The standard deviation of the confetti melanin brightness

**Table 2:** The *p*-values and mean ± SD of the significant features used to identify lesions in the denoising images.

Feature	Image Denoising	Perilesional Skin (Mean ± SD)	Lesion (Mean ± SD)	*p*-Value
*C_distance_mean*	Before	206.1 ± 17.4	200.3 ± 14.0	0.0502
	After	200.0 ± 18.5	193.5 ± 15.9	0.0402 *

* *p*-value < 0.05 shows statistically significant difference.

**Table 3:** The *p*-values and mean ± SD of the significant features used to identify lesions in the subset without the SC-DnCNN.

Layer	Feature	Image Denoising	Perilesional Skin (Mean ± SD)	Lesion (Mean ± SD)	*p*-Value
Stratum spinosum	*C_distance_mean*	Before	206.4 ± 12.9	194.3 ± 11.4	0.0036 *
		After	198.1 ± 12.9	185.3 ± 13.3	0.0032 *
	*C_distance_SD*	Before	103.7 ± 6.8	98.8 ± 6.0	0.0202 *
		After	101.1 ± 7.4	96.2 ± 6.4	0.0312 *
Dermal–epidermal junction	*G_density*	Before	5.343 ± 1.123	5.865 ± 1.124	0.1393
		After	4.905 ± 0.851	5.484 ± 0.984	0.0426 *

* *p*-value < 0.05 shows statistically significant difference.

## Data Availability

The data presented in this study are available on request from the corresponding author. The data are not publicly available due to privacy and ethical restrictions.
